# Functional Peptides from Yak Milk Casein: Biological Activities and Structural Characteristics

**DOI:** 10.3390/ijms25169072

**Published:** 2024-08-21

**Authors:** Wen Wang, Qi Liang, Baotang Zhao, Xuhui Chen, Xuemei Song

**Affiliations:** Functional Dairy Products Engineering Laboratory of Gansu Province, College of Food Science and Engineering, Gansu Agricultural University, Anning District, Lanzhou 730070, China; 13118274282@163.com (W.W.); zhaobaotang@126.com (B.Z.); cxuhui1992@163.com (X.C.); songxm@gsau.edu.cn (X.S.)

**Keywords:** yak milk casein, degradation peptides, functional activity, peptide structure, protein characterization

## Abstract

The average content of casein in yak milk is 40.2 g/L. Casein can be degraded by enzymatic digestion or food processing to produce abundant degradation peptides. International researchers have studied the degradation peptides of yak milk casein by using multiple techniques and methods, such as in vitro activity tests, cellular experiments, proteomics, bioinformatics, etc., and found that the degradation peptides have a wide range of functional activities that are beneficial to the human body, such as angiotensin-converting enzyme (ACE) inhibitory, antioxidant, anti-inflammatory, antidiabetic, antimicrobial, anticancer, and immunomodulatory activities, etc., and it has been proved that the types and strengths of functional activities are closely related to the structural characteristics of the peptides. This paper describes the characteristics of yak milk proteins, the functional activities, and mechanism of action of degraded peptides. Based on the types of functional activities of yak milk casein degradation peptides, we classified and elucidated the effects of structural factors, such as peptide molecular weight, peptide length, amino acid sequence, physicochemical properties, electrical charge, hydrophobicity, spatial conformation, chain length, and the type of enzyme on these activities. It reveals the great potential of yak milk casein degradation peptides as functional active peptide resources and as auxiliary treatments for diseases. It also provides important insights for analyzing yak casein degradation peptide activity and exploring high-value utilization.

## 1. Introduction

Bioactive peptides function as active agents that positively influence organismal health. Among these, milk-derived casein stands out as a pivotal source, where bioactivity manifests when casein peptide sequences remain intact and are primarily liberated through enzymatic processes (gastrointestinal digestion, proteolytic enzymes) and food processing methods (cooking, fermentation, ripening) [1]. Notably, diverse bioactive peptides have been identified in caseins sourced from various milks, including yak [2], cow [3], goat [4], sheep [5], buffalo [6], camel [7], and horse [8]. Initially reported in 1950, casein-degrading peptides were observed to enhance vitamin D-independent calcification in rachitic infants [9]. Subsequent investigations revealed their capacity to down-regulate cholesterol synthesis pathways, yielding cholesterol-lowering effects [3]. Furthermore, these peptides exhibit potential in regulating blood pressure by inhibiting angiotensin-converting enzyme (ACE) activity [10]. This evidence underscores milk-derived casein peptides’ therapeutic potential in disease management, characterized by high biological activity, low toxicity, and efficient metabolism compared to conventional pharmaceuticals. The mean casein concentration in yak milk surpasses that of cow milk by a factor of 1.5 [11]. The rich casein content has garnered increasing interest from scholars, as yak milk casein degradation peptides have demonstrated an array of beneficial properties, including ACE-inhibitory, antioxidant, anti-inflammatory, antidiabetic, antibacterial, anticancer, and immunomodulatory activities [12].

The molecular weight of peptides derived from yak milk casein degradation generally falls below 6000 Da, comprising an average of 2–20 amino acids. Peptides with lower molecular weights are more readily absorbed due to their smaller size. Various structural factors, such as molecular weight, peptide length, amino acid sequence, physicochemical properties, charge, hydrophobicity, spatial conformation, and chain length have been identified as influencing the functional characteristics of these peptides [2,13]. For instance, antibacterial peptides typically exhibit a hydrophobic amino acid content of around 50%, with specific structural domains that bind particular amino acids to enhance their activity. Moreover, antibacterial peptides typically carry a net charge ranging from 0 to +7 [2,14,15]. In recent years, the global adoption of molecular docking in bioinformatics has significantly facilitated the study of conformational relationships among peptides. This technology provides a streamlined approach to pinpointing specific ligand–receptor interactions, conducting detailed analyses of protein–peptide complexes and identifying active sites crucial for peptide–enzyme interactions. Such investigations delve into the functional activities and molecular mechanisms of peptides, including the role of hydrogen bonding, acknowledged as one of the most robust intermolecular forces contributing to the strength and structural integrity of protein–ligand complexes [16].

China is home to the largest number of yak species globally, with the yak population on the Tibetan Plateau constituting over 90% of the world’s total yak population [17]. The specific ecological conditions of the Tibetan Plateau and the characteristics of yaks themselves endow yak milk with several notable advantages. It is highly concentrated, rich in protein, and possesses natural and environmentally friendly attributes. Additionally, yak milk exhibits numerous beneficial properties for human health. Its ecological, economic, and resource value ranks just below that of cow’s milk. According to statistical data, China annually produces over 1.2 million tons of raw yak milk and approximately 140,000 tons of yak milk products, including cheese, yogurt, Qula, milk powder, and yak milk [11]. However, a comprehensive investigation into the structure–activity relationship of yak milk casein degradation peptides is notably lacking. This review focuses on elucidating the protein properties of yak milk, the mechanisms governing the action of its casein degradation peptides, and correlating their functional activities with corresponding structural attributes. This analysis aims to offer insights for screening, predicting functional activities, characterizing, developing, and exploring yak milk casein degradation peptides in depth.

## 2. Types and Contents of Proteins in Yak Milk

The protein composition of yak milk primarily consists of caseins (α_s1_-casein, α_s2_-casein, β-casein, and κ-casein) and whey proteins (α-lactalbumin, β-lactoglobulin, serum albumin, lactoferrin, and immunoglobulin). Table 1 illustrates the types and quantities of proteins found in various milk types. Yak milk has an average casein content of 40.2 g/L, predominantly comprising β-casein (>45%) and α_s1_-casein (40%), compared to goat milk, the levels of these four types of casein are similar, except for α_s1_-casein, where yak milk shows higher content than both cow and buffalo milk [18]. Caseins facilitate the formation of soft solids in the stomach, particularly beneficial for infant nutrition [11], and exhibit enhanced mineral-binding properties that aid in alleviating micronutrient deficiencies and exert immunomodulatory effects [19]. Whey proteins are readily digestible and absorbable, finding extensive use in sports nutrition and infant formula. Yak milk exhibits a notably high total content of whey proteins, with β-lactoglobulin, serum albumin, and lactoferrin levels surpassing those found in other milk types. β-lactoglobulin, in particular, constitutes approximately 65% of the total whey protein content in yak milk. Overall, yak milk boasts higher levels of both casein and whey proteins compared to other milks, thereby establishing a robust foundation for its rich functional properties. However, current research on bioactive peptides derived from yak milk predominantly focuses on casein proteins, with less emphasis on whey proteins.

In the preliminary work of this group, 30 female black yaks from the Gannan Tibetan Autonomous Prefecture, Gansu Province, were employed as research subjects. It was observed that both seasonal variation and parity significantly influenced the protein content of yak milk, thereby affecting the casein content (Table 2). Seasonal fluctuations impact the nutrient composition of pasture grasses. Specifically, the period from October to December represents the pre-grazing and dry grass period in Gannan, during which yaks experience insufficient nutrient intake. Consequently, while milk yield is lower, the protein content is elevated. Conversely, from July to August, during the green grass and abundant grass periods, yaks receive adequate nutrition, leading to increased milk yield and a stabilization of protein content. Notably, the protein content of Gannan black yak milk peaks in December. Parity also plays a crucial role in the nutrient absorption from pasture, with larger parity correlating with increased milk yield. The protein content of yak milk is highest at the third parity, showing an increase from the first to the third parity, but a decrease from the third to the fifth parity. Therefore, the casein content of yak milk is maximal at the third parity and at twelve months.

## 3. Functional Activities of Degraded Peptides from Different Milk Sources

In combating health disorders, pharmacological treatments using drugs have proven effective but are accompanied by adverse side effects on the human body. In response, bioactive peptides derived from milk-derived proteins have been explored as adjunct therapies for regulating organismal health. International researchers have used cell culture, animal models, and in vitro methods from cow milk, buffalo milk, camel milk, and goat milk and have found that peptides exhibit nine functional activities, including anti-osteoporotic [22], anti-inflammatory [6], antibacterial [23], antioxidant [24], cholesterol-lowering [3], ACE-inhibitory [25], anticancer [26], antidiabetic [3], and immunomodulatory [27] (Table 3). While peptide sequences for most activities have been elucidated for further investigation, antibacterial and anticancer activities have only been observed in hydrolysates of milk-derived proteins, with specific peptide sequences yet to be determined.

**Figure 1:**
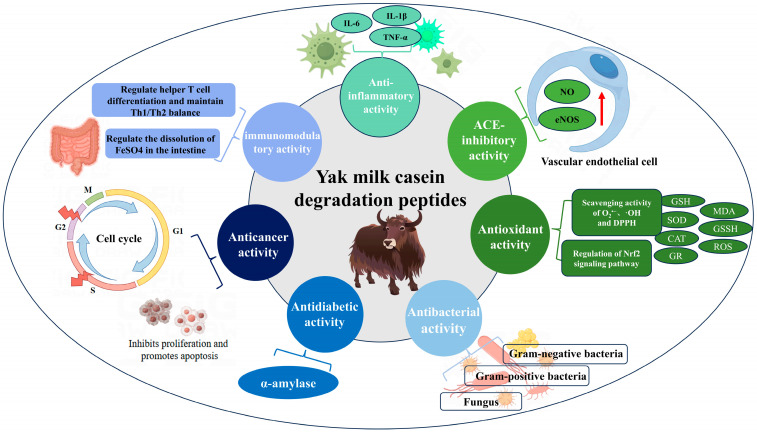
Potential activity and mechanism of yak milk casein degradation peptides.

Figure 1 illustrates seven functional activities and their corresponding mechanisms of action attributed to ACE-inhibitory, antioxidant, anti-inflammatory, antidiabetic, antibacterial, anticancer, and immunomodulatory activities identified in yak milk casein degradation peptides by current researchers. The ACE-inhibitory peptide derived from yak milk exhibits affinity towards ACE, competing for substrate binding to lower blood pressure. Specifically, the yak milk ACE-inhibitory peptide KYIPIQ enhances nitric oxide (NO) synthesis and endothelial nitric oxide synthase (eNOS) expression in human vascular endothelial cells, potentially aiding in hypertension treatment and related conditions [28]. Moreover, the antioxidant peptide from yak milk casein regulates the Nrf2 pathway protein expression, inhibits apoptosis, scavenges superoxide anion (O_2_^•−^), hydroxyl radical (·OH), and 1,1-diphenyl-2-picryl-hydrazyl (DPPH) in vitro, and enhances glutathione (GSH) capacity, catalase (CAT) activity, glutathione reductase (GR) and superoxide dismutase (SOD) activity. Additionally, it reduces malondialdehyde (MDA), oxidized glutathione (GSSG), and reactive oxygen species (ROS) levels, thereby demonstrating robust antioxidant properties. In the investigation of yak milk casein anti-inflammatory peptide, Mao et al. [29] found that yak milk casein anti-inflammatory peptide treats inflammation by inhibiting the anti-inflammatory factors interleukin-6 (IL-6), interleukin-1β (IL-1β), and tumor necrosis factor alpha (TNF-α). Milk-derived antidiabetes peptides could treat diabetes by inhibiting dipeptidyl peptidase-IV (DPP-IV), α-glucosidase, and α-amylase, but only the antidiabetes peptides RPKHPIK (RK7) and KVLPVPQ (KQ7), which could inhibit α-amylase, have been identified in yak milk casein [16]. Antibacterial peptides derived from yak milk casein exhibit inhibitory activity against Gram-positive bacteria, Gram-negative bacteria, and certain fungi. These peptides have shown significant effectiveness against Escherichia coli and Staphylococcus aureus. The anticancer peptide TPVVVVVPPFL from yak milk casein inhibits the proliferation of MCF7 and MDA-MB-231 cells, inducing apoptosis by arresting the cell cycle [30]. Immunomodulatory peptides from yak milk casein modulate physiological functions by aiding in T cell differentiation, regulating the Th1/Th2 balance and modulating FeS0_4_ solubilization in the intestine. Further exploration may reveal additional multifunctional activities in yak milk casein, such as potential roles in osteoporosis prevention and cholesterol reduction, which currently lack sufficient research documentation.

## 4. Functional Activity and Structural Characterization of Yak Milk Casein Degradation Peptides

### 4.1. Structure–Activity Relationship of ACE-Inhibitory Peptides

**Figure 2:**
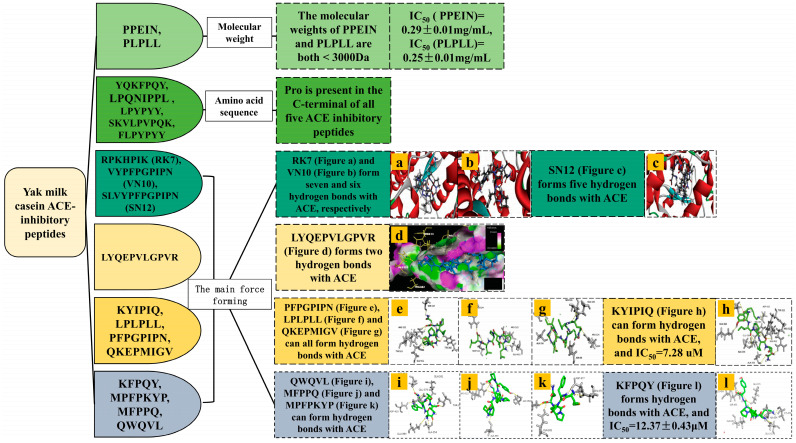
Structural characterization of ACE-inhibitory peptide of yak milk casein.

ACE is a pivotal component of the renin–angiotensin–aldosterone system, intricately associated with blood pressure regulation and cardiovascular health [31]. Clinical studies have demonstrated that ACE inhibitors, such as captopril, lisinopril, and enalapril, significantly reduce cardiovascular mortality rates by up to 12%. In recent years, an increasing number of antihypertensive peptides have been identified within food proteins. Notably, yak milk casein degradation peptides (Table 4, Figure 2) encompass several peptides with such bioactive properties. For instance, the peptide KYIPIQ exhibits potent ACE-inhibitory activity with an IC_50_ value of 7.28 uM, suggesting its potential as an adjunctive therapy for hypertension [28,32].

Natural ACE-inhibitory peptides typically consist of fewer than 12 amino acid residues, with most exhibiting a molecular weight below 3000 Da. It has been observed that the smaller the molecular weight of the hydrolyzed product, the stronger its ACE-inhibitory activity [39]. Mao et al. conducted the hydrolysis of yak milk casein using alcalase and subsequently purified and isolated two ACE-inhibitory peptides with low molecular weights: PPEIN (550 Da) and PLPLL (566.4 Da) [33]. These peptides demonstrated IC_50_ values of 0.29 ± 0.01 mg/mL and 0.25 ± 0.01 mg/mL, respectively. Analogously, Li et al. discovered four ACE-inhibitory peptides characterized by their low molecular weight and short peptide chains, derived from whey protein [40].

The ACE-inhibitory activity of peptides is closely related to the presence of hydrophobic amino acids such as Pro, Ile, Leu, and Trp. The residues close to the three C-terminal positions are hydrophobic amino acids favoring ACE binding, and the presence of C-terminal Pro enhances the ACE-inhibitory potential of the peptides by increasing the hydrophobic interactions in the catalytic site of ACE [34]. Jiang et al. used neutrase to isolate five ACE-inhibitory peptides from the hydrolyzed product of yak milk casein, all of which had Pro at the C-terminus [34].

ACE is a metalloprotease with a zinc ion Zn^2+^ interacting with His383, His387 and Glu411 with three active sites including S1 (Ala354, Glu384 and Tyr523), S1′ (Glu162), and S2 (Gln281, His353, Lys511, His513, Tyr520 and His523), which are not only essential for ACE activity but also serve as the main binding targets for ACE-inhibiting drugs. Most of the ACE inhibitors and bioactive peptides interact with and inhibit the C and N structural domains of sACE and tACE through the formation of hydrogen bonding and hydrophobic interactions at the S1, S2, and S1′ sites of the ACE or by directly binding to catalytic Zn^2+^ [32]. Yang et al. discovered that yak milk cheese bitter peptides RK7, VYPFPGPIPN (VN10), and SLVYPFPGPIPN (SN12) form seven, six, and five hydrogen bonds, respectively, and that both RK7 and VN10 interacted with the ACE major active sites, Glu411 and His387, along with other ACE amino acid residues. Specifically, RK7 and VN10 interacted prominently with the major active sites of ACE, Glu411, and His387 [10]. Abedin et al. enzymatically hydrolyzed yak hard chhurpi using two enzymes, resulting in the isolation of the ACE-inhibitory peptide LYQEPVLGPVR. This peptide forms hydrogen bonds with the ACE major active sites Glu411 and Tyr523, as well as hydrophobic interactions with His353, His383, and His513 [35]. Lin et al. employed multiple enzymes to hydrolyze Qula casein, yielding eight ACE-inhibitory peptides. Among them, PFPGPIPN forms hydrogen bonds with ACE’s main active sites Tyr523 and Glu411. KYIPIQ forms hydrogen bonds with Ala354, Glu384, Tyr523, Glu162, His383, and Glu411. Both LPLPLL and QKEPMIGV form hydrogen bonds with Glu411. QWQVL forms hydrogen bonds with Ala354, Glu384, and Gln281. KFPQY forms hydrogen bonds with Ala354, Gln281, and Glu162. MFPPQ forms hydrogen bonds with Gln281 and Lys511. MPFPKYP forms hydrogen bonds with Ala354. KYIPIQ exhibited the highest ACE-inhibitory activity when compared to captopril used as a positive control [28,36,37,38]. Baba et al. extracted two ACE-inhibitory peptides from camel whey: ACEPAGNFLP, which formed hydrogen bonds with Ala354, Tyr523, Gln281, and Lys511; and FCCLGPVP, which interacted with Ala354 and Tyr523. these binding sites are basically the same as those of the binding site of ACE-inhibitory peptides from yak milk casein [41]. Chamata et al. explored the ACE-inhibitory potential of whey-derived peptides by comparing their binding sites with known ACE inhibitors. Sampatrilat is a potent ACE inhibitor, and the peptides IIAE, LIVTQ, and LVYPFP share three interactions with Sampatrilat: IIAE interacts with Gln259, LVYPFP interacts with His331, and IIAE, LIVTQ, and LVYPFP all interact with Thr358. Additionally, IIAE and lisinopril all interact with Asp140, while LIVTQ interacts with Ala332, a residue shared by lisinopril and enalapril [42].

Most of the ACE-inhibitory peptides from various milk sources are small-molecule peptides containing hydrophobic amino acids that interact with Zn^2+^ and specific amino acid residues within the S1, S2, and S1 pockets, thereby forming intermolecular forces, which may be one of the reasons for the competitive or mixed competitive mode of inhibitory activity. ACE-inhibitory activity is predominantly influenced by both the primary and secondary structures of these peptides. For small peptides possessing 2–8 amino acids, the amino acid sequence is the main determinant of their ACE-inhibitory activity. Factors such as peptide length, molecular weight, and amino acid composition elucidate the primary structure of ACE-inhibitory peptides, whereas for larger peptides exceeding nine amino acids, the secondary structure can be formed and influence their activity.

### 4.2. Structure-Activity Relationship of Antioxidant Peptides

**Figure 3:**
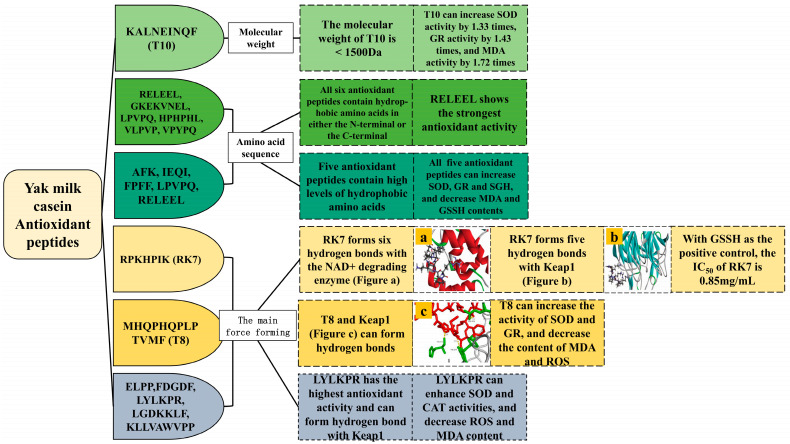
Structural characterization of antioxidant peptides from yak milk casein.

Oxidation is one of the main causes of several chronic diseases, during which harmful ROS are produced. Typically, the body’s antioxidant system neutralizes these radicals to maintain homeostasis. However, when this scavenging process is insufficient, oxidative stress ensues, potentially leading to conditions such as arthritis, cancer, and age-related ailments [43]. In contrast to macromolecular proteins and synthetic antioxidant medications, natural antioxidant peptides sourced from dairy products are perceived to offer advantages in terms of safety, non-toxicity, and enhanced bioavailability. Yang et al. identified the antioxidant peptide KQ7 (IC_50_ = 0.85 mg/mL) from yak milk cheese with a slightly lower activity than that of GSSG (IC_50_ = 0.65 mg/mL) [44]. Other yak milk casein antioxidant peptides and their structural characteristics are as follows (Table 5, Figure 3).

Antioxidant peptides sourced from food typically range in size from 500–1500 Da and comprise fewer than 20 amino acids [46]. Yang et al. employed trypsin and pepsin for the hydrolysis of yak milk residues, identifying KALNEINQF (T10) with a molecular weight of 1076.20 Da as the peptide exhibiting the strongest antioxidant activity in their study. Upon the treatment of human umbilical vein endothelial cells (HUVEC) with peptide T10, compared with the H_2_O_2_-injured group, the SOD activity was significantly increased by 1.03 times, 1.1 times, and 1.33 times, the GR activity was significantly increased by 1.11 times, 1.30 times, and 1.43 times, MDA was also decreased by 1.41 times, 1.54 times, and 1.72 times [45].

Peptides containing hydrophobic amino acids such as Trp, Tyr, Phe, or Lys exhibit potent antioxidant activity, which is further enhanced when these amino acids are located at the N-terminal or C-terminal ends of the peptides [4,37]. Liu et al. identified six antioxidant peptides from yak milk casein, characterized by the presence of hydrophobic amino acids at either terminal end. Among these, RELEEL demonstrated the highest antioxidant potency, displaying significant scavenging activities against O_2_^•−^ (IC_50_ = 0.52 mg/mL) and ·OH radicals (IC_50_ = 0.69 mg/mL) [46]. Wu et al. investigated five antioxidant peptides derived from yak milk casein, highlighting LPVPQ and RELEEL as particularly effective due to their composition of hydrophobic amino acids such as Pro, Leu, and Val. RELEEL notably reduced the MDA content (0.062 ± 0.0004 U/10^4^ cells) and enhanced the GR activity (61.17 ± 2.479 μg/10^6^ cells), while the peptide LPVPQ significantly reduced the GSSG content (0.74 ± 0.26 μg/10^6^ cells) and boosted the SOD activity (1.17 ± 0.016 U/10^4^ cells) [47]. Ibrahim et al. and Cui et al. also observed that antioxidant peptides derived from camel milk protein and cow milk protein, respectively, contain hydrophobic amino acids, aligning with the findings on yak milk casein peptides. Additionally, their studies indicated that casein-derived antioxidant peptides possess a higher proportion of hydrophobic amino acids compared to whey proteins [50,51].

The antioxidant peptides derived from yak milk casein have the capability to interact with amino acids located within the catalytic cavities of enzymes responsible for oxidation in organisms, thereby inhibiting their activity for therapeutic purposes. Yang et al. identified the antioxidant peptide RK7 from yak milk cheese. RK7 forms six and five hydrogen bonds with the NAD+ degradation enzyme and Keap1, respectively. Notably, the amino acid residues Arg140 and Asp147 of the NAD+ degradation enzyme, as well as Ser390 and Asp394 of Keap1, were identified as crucial active sites for RK7 binding [44]. Qin et al. enzymatically hydrolyzed yak milk residues using trypsin and pepsin, resulting in the identification of the antioxidant peptide MHQPHQPLPTVMF (T8) from the hydrolysate. T8 was found to form hydrogen bonds with amino acid residues His575, Phe577, and Arg336 of Keap1, indicating its robust stability in binding to Keap1. Furthermore, compared to the control group, T8 significantly enhanced SOD and GR activities in HUVEC cells and reduced the MDA and ROS contents [48]. Jiang et al. identified five antioxidant peptides from yak milk fermented with Lactobacillus plantarum JLAU103, and the antioxidant activity of LYLKPR was the most pronounced. LYLKPR formed hydrogen bonds with the Keap1 protein amino acid residues Leu365, Asn382, Thr481, and Arg483 and formed Ala556, Leu365, and Ile46 hydrophobic interactions. LYLKPR significantly increased the SOD and CAT activities and decreased the ROS and MDA contents in HT-22 cells compared to the control [49]. Different milk-derived antioxidant peptides have different active sites for binding to Keap1 protein. Tonolo et al. used trypsin to extract three novel antioxidant peptides from fermented milk casein, all of which successfully docked with the Keap1 protein. APSFSDIPNPIGSENSE formed hydrogen bonds with Ser363, Arg415, Tyr572, and Arg336. QGPIVLNPWDQVKR formed hydrogen bonds with Asn382, Arg380, Gln530, Thr576, His575, Asn387, Arg380, His432, Tyr525, Arg415, and Ser508. NTVPAKSCQAQPTTm (which has an oxidized Met at position 15) formed hydrogen bonds with Asn38, Gly509, Tyr572, Arg336, Arg380, Arg415, Ser602, Gly433, Ile435, and Thr576 [52].

Most of the antioxidant peptides derived from various milk sources contain hydrophobic or specific amino acids. However, their binding sites on Keap1 vary, likely influenced by the amino acid composition and peptide sequences of these active peptides. The antioxidant activity of these peptides is notably affected by factors such as the protein source, isolation conditions, degree of hydrolysis, and hydrolysis process. Antioxidants are closely associated with anti-inflammatory, anticancer, and immunomodulatory properties, where ROS plays a pivotal role in the pathogenesis of these diseases. Further research into developing yak milk casein-derived peptides with potent antioxidant properties holds promise for enhancing human health.

### 4.3. Structure–Activity Relationship of Anti-Inflammatory Peptides

Inflammation serves as a primary mechanism by which the body defends itself against microbial intrusions. When inflammation occurs, macrophages secrete pro-inflammatory cytokines and NO, contributing to the onset and propagation of the inflammatory response. This process can ultimately give rise to conditions such as diabetes, neurodegenerative disorders, Parkinson’s disease, cardiovascular ailments, and various cancers in humans [53]. International researchers have isolated anti-inflammatory peptides from foodstuffs, including walnuts and buffalo milk. These peptides exhibit promising attributes in the prevention of inflammation and the mitigation of chronic illnesses. Incorporating anti-inflammatory peptides into the diet emerges as a novel therapeutic approach to combat inflammation. For instance, GPETAFLR, a newly identified anti-inflammatory peptide derived from lupine protein isolate, aids in the prevention of chronic inflammation [54]. The anti-inflammatory peptides of yak milk casein are shown in Table 6.

The molecular weight of anti-inflammatory peptides is typically less than 1000 Da. Peptides of this size range from 2 to 20 residues and are more readily absorbed by the intestine and able to penetrate cell membranes compared to larger peptides. They commonly feature hydrophobic amino acids such as Val, Ile, and Pro, as well as positively charged residues including His, Arg, and Lys [55]. Bamdad et al. utilized alcalase to enzymatically hydrolyze β-lactoglobulin, resulting in the production of eight distinct anti-inflammatory peptides ranging from 6 to 13 amino acids in length. The hydrolysate demonstrated significant reductions in the NO levels and pro-inflammatory cytokines such as TNF-α and IL-1β. Approximately 38% of the identified peptides exhibited notable hydrophobic characteristics, enriched with aromatic residues, thereby enhancing their anti-inflammatory efficacy [55]. Moreover, Zhao et al. identified two hydrophobic anti-inflammatory peptides, namely DQPFFHYN (DN8) and YSPFSSFPR (YR9), derived from Binglangjiang buffalo milk sourced from Tengchong, Yunnan Province, China. The YR9 peptide terminates with an Arg residue, and both peptides exhibited significant inhibitory effects on NO secretion, TNF-α, IL-6, and the expression of inducible nitric oxide synthase (iNOS) in LPS-stimulated RAW264.7 cells [56]. Additionally, Mao et al. investigated antioxidant peptides derived from yak milk casein, which exhibit anti-inflammatory properties. The hydrolysis of yak milk casein by alcalase in the presence of LPS resulted in a significant dose-dependent inhibition of IL-6, IL-1β, and TNF-α, with effective concentrations ranging from 0.125 to 1.0 mg/mL [29].

The anti-inflammatory activity of yak milk casein degradation peptides has been less studied internationally. Furthermore, peptide sequences have not yet been identified, leaving open the exploration of whether these peptides exhibit structural characteristics typical of anti-inflammatory peptides found in other milk sources. For instance, there is scant investigation into their impact on key inflammatory enzymes such as iNOS and cyclooxygenase-2 (COX-2).

### 4.4. Structure–Activity Relationship of Antidiabetic Compounds

**Figure 4:**
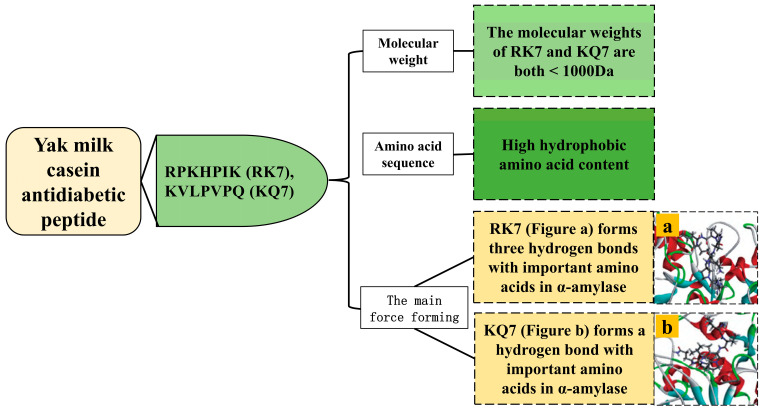
Structural characterization of antidiabetic compounds from yak milk casein.

The incidence of Type 2 diabetes mellitus (T2DM) is escalating globally, with China currently leading the world in diabetes cases, affecting over 94 million individuals [57]. While acarbose is widely used by patients to manage T2DM, its prolonged usage may induce gastrointestinal side effects. Recently, peptides sourced from natural food proteins, renowned for their antidiabetic attributes, have garnered significant research attention. According to Li et al., an antidiabetic peptide, RK7 (IC_50_ = 0.45 mg/mL), has been isolated from yak milk cheese (Table 7, Figure 4). Although slightly less potent than acarbose (IC_50_ = 0.36 mg/mL), RK7 exhibits considerable therapeutic promise for T2DM [13]. Antidiabetic peptides primarily function through two mechanisms: inhibiting DPP-IV, which regulates insulin secretion, and suppressing α-glucosidase or α-amylase to regulate or delay glucose absorption in the small intestine, thereby reducing postprandial hyperglycemia [57,58]. Notably, Mojica et al. have successfully extracted antidiabetic peptides from black beans, demonstrating the inhibitory effects on DPP-IV, α-glucosidase, and α-amylase [59].

Peptides with a molecular weight less than 1000 Da exhibit enhanced inhibition of α-amylase. Li et al. identified two small molecular weight α-amylase inhibitory peptides, RK7 (875.07 Da) and KQ7 (779.98 Da), from yak milk cheese [13].

Hydrophobic amino acids such as Ala, Leu, Phe, Val, Pro, and Gly, as well as high molecular weight amino acids with aromatic rings, including Arg, Phe, Trp, and Tyr, play crucial roles in α-amylase inhibition [13]. The α-amylase inhibitory peptides RK7 (possessing Pro and Ile) and KQ7 (possessing Val, Pro, and Leu) both possess high hydrophobic amino acid content [13].

The active sites of α-amylase encompass Trp58, Trp59, Tyr62, Asp96, His101, Arg195, Asp197, Glu233, Asp236, His299, Asp300, and His305 [57]. Both RK7 and KQ7 form intermolecular forces with His305, Glu233, Trp59, and Trp58 in α-amylase, and binding to Glu233 enhances their inhibitory activity against α-amylase [13]. The α-amylase binding site for antidiabetic peptides from yak milk casein resembles that found in other milk sources. Mudgil et al. utilized molecular docking techniques, the two most potent α-amylase inhibitory peptides were identified among four probiotic-derived fermented milk products. One of these, IMEQQQTEDEQQDK from fermented camel milk, forms nine hydrogen bonds with the enzyme’s key active site residues Asp300 and His305. Similarly, the fermented goat milk-derived peptide DQHQKAMKPWTQPK interacts via hydrogen bonding with eight different residues on the enzyme, including Trp58, Trp59, Tyr62, Asp300, and His305 [60].

Currently, international researchers have noted similarities in the antidiabetic properties of yak milk casein compared to other dairy sources. These similarities arise from the presence of hydrophobic amino acids and aromatic ring macromolecular amino acids, which interact with the primary active sites of α-amylase. However, research into the inhibitory effects and structural characteristics of these peptides against DPP-IV and α-glucosidase enzymes is lacking. Therefore, further investigation is necessary to explore the potential antidiabetic peptides isolated from yak milk casein. The largest number of peptides reported for the adjuvant treatment of T2DM includes Glucagon-like peptide-1 (GLP-1), which has emerged as a significant research focus. Studies indicate that long-term administration of GLP-1 in T2DM treatment results in substantial improvements in patient health and a reduction in overall treatment costs. This suggests that the use of peptides for adjuvant therapy holds great promise [61].

### 4.5. Structure–Activity Relationship of Antibacterial Peptides

**Figure 5:**
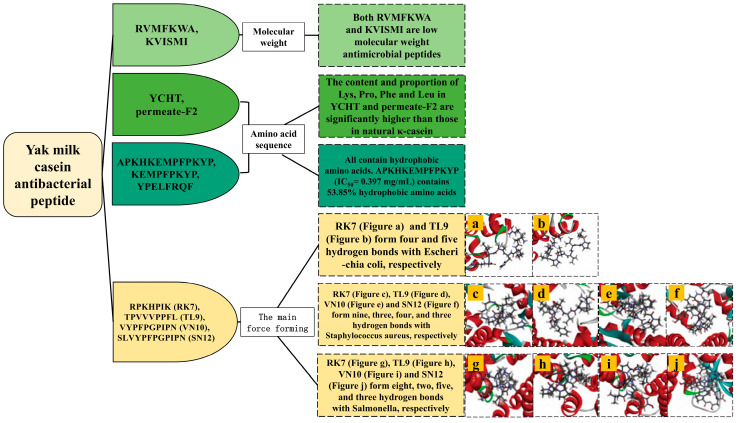
Structural characterization of antibacterial peptides from yak milk casein.

Antibiotic-resistant Gram-negative and Gram-positive bacteria represent the primary etiology of bacterial infections, exacerbated by the widespread use of antibiotics, which has precipitated the emergence of resistance phenomena and the genesis of superbugs [62]. Antibacterial peptides, owing to their biocompatibility and biodegradability relative to small molecule antibiotics, exhibit reduced toxicity. Furthermore, their distinct mechanism of membrane disruption mitigates the propensity for inducing bacterial resistance. Investigation into the antibacterial efficacy and structural attributes of peptides derived from yak milk casein degradation holds promise for advancing antibiotic discovery endeavors (Table 8, Figure 5).

Antibacterial peptides typically consist of fewer than 50 amino acids [66]. Pei et al. isolated two low-molecular-weight antibacterial peptides, namely RVMFKWA (937.17 Da) and KVISMI (689.91 Da), which showed inhibitory effects against Bacillus subtilis, Staphylcoccus aureus, Listeria innocua, Escherichia coli, Enterobacter cloacae, and Salmonella paratyphi. KVISMI also inhibited some fungi (Candida albicans and Saccharomyces cerevisiae) [63].

The efficacy of antibacterial peptides is significantly influenced by the presence of hydrophobic and basic residues. Typically, these peptides comprise approximately 50% hydrophobic amino acids and exhibit high levels of Arg, Pro, Trp, and His. The hydrophobicity of antibacterial peptides plays a crucial role in their activity [14]. Cheng et al. demonstrated that κ-casein derived from yak milk casein through trypsin hydrolysis (YCHT) and its isolated fraction (permeate-F2) had markedly higher contents and ratios of Ly), Pro, Phe, and Leu compared to natural κ-casein. This compositional difference was associated with increased inhibitory activities of YCHT and permeate-F2 against Escherichia coli compared to natural κ-casein [64]. Additionally, Zhang et al. identified three antibacterial peptides from yak milk casein using the flavozyme treatment. Among these, the peptide APKHKEMPFPKYP (IC_50_ = 0.397 mg/mL) contains 30.77% basic amino acids and 53.85% hydrophobic amino acids, demonstrating significant inhibitory activity against Staphylococcus aureus [65].

Bioinformatics techniques effectively resolved the differences in the bacteriostatic activities of antibacterial peptides, as well as the amino acid and positional information of their interactions with different microbial bacterial proteins. Yang et al. screened four antibacterial peptides, and RK7 and TL9 were successfully docked with Escherichia coli (four and five hydrogen bonds, respectively), Staphylcoccus aureus (nine and three hydrogen bonds, respectively), and Salmonella (eight and two hydrogen bonds, respectively). In addition, VN10 and SN12 were successfully docked with Staphylcoccus aureus (four and three hydrogen bonds, respectively) and Salmonella (five and three hydrogen bonds, respectively) [2]. In other dairy sources, international researchers have adopted different enzymes to study the activity of antibacterial peptides. GlcN-6-P synthetase is an important component of bacteria. Singh et al. hydrolyzed the whey protein of Himalayas goat breed “Gaddi” using alcalase and flavozyme to produce the antibacterial peptide DDSPDLPK, which formed hydrogen bonds with the amino acid residues Asp548, Tyr576, Asp474, and Glu569 of GlcN-6-P synthase [67]. MurD ligase is a peptide ligase that is involved in the formation of microbial cell walls. Iram et al. utilized the Lactobacillus rhamnosus (C25) fermentation of goat milk to generate antibacterial peptides with potent bacteriostatic effects against both Gram-positive and Gram-negative bacteria. Specifically, the peptide KSFCPAPVAPPPPT formed hydrogen bonds with MurD ligase amino acid residues Phe419, Leu416, and Ala77, while the peptide IGHFKLIFSLLRV interacted with MurD ligase amino acid residues Leu15, Thr16, Asn138, Arg37, Gly73, Asn421, and Lys420 [68].

Milk-derived antibacterial peptides typically exhibit high molecular weights and lengthy peptide sequences, often not exceeding 50 amino acids. They predominantly consist of hydrophobic amino acids and possess a net charge ranging from 0 to +7, essential criteria for their antibacterial activity. The interactions of antibacterial peptides with GlcN-6-P synthase and MurD ligase were investigated in goat and sheep milk and were found to be directly related to the antibacterial activity of the peptides, which provided important inspiration and reference for the study of yak milk casein antibacterial peptides. Although there are many types of antibacterial peptides, most of them have long sequences, complicated extraction processes, and high synthesis costs. Consequently, there is a burgeoning interest in developing short antibacterial peptides characterized by low production costs and potent antibacterial properties, representing promising avenues for future research.

### 4.6. Structure–Activity Relationship of Anticancer Peptides

Cancer poses a substantial impediment to increasing human longevity. In 2020, the World Health Organization reported 19.3 million new cases of cancer and 10 million cancer-related deaths worldwide. Projections indicate a future rise to 27.5 million new cases annually by 2040 [69]. Peptides that exhibit minimal toxicity to normal tissues are rapidly evolving as anticancer agents, with peptides released from yak milk casein possessing antioxidant and anti-inflammatory activities, theoretically suggesting anticancer properties (Table 9).

Gu et al. employed trypsin and alkaline protease for the hydrolysis of yak milk casein, yielding anticancer peptides such as TPVVVVVPPFL, VAPFPEVFGK, and NQFLPYPY. These peptides are predominantly composed of amino acid sequences commonly identified in casein-derived anticancer peptides, including Pro, Val, Phe, Leu, Lys, and Gln. Moreover, the amino acid composition is largely hydrophobic. There is significant hydrophobicity, and the relationship between hydrophobicity and anticancer effects is positively correlated [30].

Gu et al. investigated the impact of the physicochemical properties of yak milk casein degradation peptides on their anticancer efficacy [30]. Meanwhile, Murali et al. identified two promising anticancer peptides through the pepsin hydrolysis of camel whey proteins. AHLEQVLLR was found to engage in hydrogen bonding with amino acid residues Arg516 and His538 of Polo-like kinase 1, whereas ALPNIDPPTVER exhibited a more pronounced binding pattern, forming hydrogen bonds with Tyr417, Tyr485, and Arg516 of Polo-like kinase 1 [70]. Currently, while international researchers have characterized the peptide sequences of yak milk casein’s anticancer peptides, structural investigations remain limited. Furthermore, research on the secondary structure of these peptides is still exploratory.

### 4.7. Structure–Activity Relationship of Immunomodulatory Peptides

In recent years, heightened attention has been directed towards the immune system, prompted by a rise in chronic and other health-related conditions. Immunomodulatory drugs currently in development are costly and exhibit adverse effects on human physiology. Cow milk-derived casein and whey proteins have been explored as potential sources for generating immunomodulatory peptides, which offer a promising avenue due to their safety profile and high bioavailability. Sütas et al. substantiated this by showcasing the in vitro immunomodulatory impacts of peptides derived from casein on human blood lymphocyte proliferation [71]. International researchers have also found immunomodulatory peptides in yak milk casein (Table 10).

International researchers have explored the immunomodulatory properties of peptides derived from yak milk casein through various approaches, including cell culture and in vitro methods. Mao et al. reported that hydrolysates generated via alcalase hydrolysis of yak milk casein exhibited heightened immunomodulatory activity, an increased degree of hydrolysis, and enhanced peptide yield. The molecular weights of isolated fractions of casein hydrolysates were consistently <1500 Da. These hydrolysates demonstrated the capability to regulate helper T cell differentiation and maintain Th1/Th2 balance, indicating potential therapeutic applications in cell-mediated immune disorders [72,73]. Furthermore, Wan et al. identified carboxylate and amide groups as the principal binding sites for Fe^2+^ within yak milk casein hydrolysates. They highlighted that peptide length and size significantly influenced the binding efficacy of the hydrolysate with Fe^2+^, and the formation of the complexes between the casein hydrolysate and divalent cations was obviously related to the electron supplying ability of the hydrolysate [74].

The immunomodulatory effects of milk-derived peptides are associated with the physicochemical properties of the amino acids, including positive charge, hydrophobicity, and chain length. Positively charged peptides have been demonstrated to bind and activate chemokine receptors on immune cells [75]. It is widely acknowledged that numerous milk-derived peptides exhibit immunomodulatory properties, a phenomenon believed to be mediated by the presence of Arg at either the C-terminal or N-terminal position. Furthermore, the structural characterization of immunomodulatory peptides derived from yak milk casein remains in the exploratory phase, indicating that this area of research is actively being developed.

## 5. Conclusions and Prospects

As the global awareness of health continues to increase, yak milk protein, noted for its distinct properties, has become a prominent focus of international research. An increasing number of novel sequences of yak milk casein degradation peptides are being unveiled, offering the advantages of low or no toxicity, fast absorption, and minimized tissue damage. This sets them apart from conventional drugs that often come with a similar level of toxicity and side effects. Recent advancements in peptide-assisted treatments for T2DM have demonstrated significant progress, with peptide therapies proving to be more cost-effective compared to traditional treatments. Despite this, the high cost of peptide synthesis remains a substantial barrier. Research has identified various functional activities in yak milk casein degradation peptides, indicating their potential in disease treatment and health regulation. However, the current body of research predominantly consists of in vitro experiments and cell culture studies, with a notable absence of clinical trials to validate these findings. This highlights a critical need for clinical research to further explore the therapeutic potential of yak milk casein degradation peptides. Currently, research has primarily focused on the antioxidant, ACE-inhibitory, and antibacterial properties of yak milk casein degradation peptides. However, investigations into their potential antidiabetic, anti-inflammatory, anticancer, and immunomodulatory activities are limited. Particularly, understanding the structural basis for the anti-inflammatory, anticancer, and immunomodulatory effects remains a significant knowledge gap, with some peptide sequences yet to be fully elucidated. Furthermore, while bioactive peptides from other milk sources have shown anti-osteoporotic and cholesterol-lowering effects, such investigations are absent for yak milk casein degradation peptides. It is essential to acknowledge that certain bioactive peptides might undergo structural alterations during digestion or processing. Therefore, future research endeavors should explore the dynamic structural changes in these peptides post-administration through in vivo studies, including molecular dynamics simulations, animal trials, and clinical models. These studies aim to unveil potential complex formations of active peptides with other molecules during processing, as well as to identify any associated adverse reactions. By elucidating unidentified peptide sequences, researching new technologies to reduce the cost of extracting and synthesizing peptides, investigating the conformational relationships underlying known activities, exploring novel functional properties, and bridging existing gaps in experimental methodologies and models, we can further unlock the therapeutic potential of yak milk casein degradation peptides. We will further explore the value of yak milk casein degradation peptides, work together in a multidisciplinary way, and promote the development and utilization of yak milk casein degradation peptides.

## Figures and Tables

**Table 1:** The types and contents of proteins in different kinds of milk [11,12,20].

Types of Proteins	Yak	Cow	Buffalo	Goat
Total casein (g/100 g)	3.43–4.58	2.40–2.80	2.70–5.00	2.30–3.80
α_S1_-casein (mg/100 g)	930–1310	806–1508	1147–1924	135–1020
α_S2_-sein (mg/100 g)	360–650	182–390	222–629	270–750
β-casein (mg/100 g)	1500–2060	728–988	1295–1702	1020–1920
κ-casein (mg/100 g)	490–850	234–520	407–592	300–570
Total whey proteins (g/100 g)	1.10	0.50–0.70	0.60–1.00	0.30–1.20
α-lactalbumin (mg/100 g)	20–170	96–150	117–303	85–250
β-lactoglobulin (mg/100 g)	340–1010	198–402	301–441	170–385
Serum albumin (mg/100 g)	20–310	36–45	2.1–35	25–110
Lactoferrin (mg/kg)	200–700	20–500	20–300	20–300
Immunoglobulins (mg/kg)	100–400	150–1000	500–1300	150–500

**Table 2:** Effects of different lactation months and parity on protein content in yak milk (%) [21].

Parity	June	July	August	September	October	November	December
1st parity	4.59 ± 0.10	4.27 ± 1.02	4.61 ± 0.21	4.39 ± 0.53	4.54 ± 0.53	4.89 ± 0.49	5.35 ± 0.41
2nd parity	4.59 ± 0.03	4.77 ± 0.89	4.20 ± 0.33	4.37 ± 0.33	5.13 ± 0.51	4.99 ± 0.84	6.07 ± 0.47
3rd parity	4.78 ± 0.12	4.85 ± 0.30	4.30 ± 0.16	4.65 ± 0.13	5.15 ± 0.51	5.11 ± 0.37	6.34 ± 1.14
4th parity	4.70 ± 0.18	4.65 ± 0.25	4.13 ± 0.30	3.84 ± 0.36	4.94 ± 0.41	4.95 ± 0.41	6.18 ± 0.38
5th parity	4.47 ± 0.23	4.42 ± 0.51	3.66 ± 0.24	4.22 ± 0.11	4.74 ± 0.38	4.76 ± 0.70	5.95 ± 0.27

**Table 3:** Functional activities of bioactive peptides from different milk sources.

Source of Bio-Active	Peptide Sequence	Model/Method	Functional Activity
Cow milk	VLPVPQK	Rat osteoblast cultures	Anti-osteoporotic
Buffalo milk casein	YFYPQL	Mice splenocytes culture	Anti-inflammatory
Camel milk	-	In vitro methods	Antibacterial
Cow milk	YFYPEL	In vitro methods	Antioxidant
Cow milk casein	VLPVPQ, VAPFPE, LQPE, TDVEN	In vitro methods	Cholesterol-lowering
Cow milk	IPP, VPP	In vitro methods	ACE-inhibitory
Buffalo and cow milk cheddar cheeses	-	Lung cancer (H-1299) cell line	Anticancer
Goat milk casein	INNQFLPYPY	In vitro methods	Antidiabetic
Buffalo colostrum whey	DMIVGPGNLQEGESEGDSQK, LGEYGFQNALIVR, CCAADDKEACFAVEGPK and so on	Macrophage culture	Immunomodulatory

**Table 4:** ACE-inhibitory activity of yak milk casein degradation peptides.

Source of Bio-Active	Used Enzymes	Peptide Sequence	Model/Method	Reference
Yak milk cheese	Pepsin and trypsin	RPKHPIK, VYPFPGPIPN, SLVYPFPGPIPN	In vitro methods and molecular docking	[10]
Yak milk casein	-	KYIPIQ	HUVECs culture	[28]
Yak milk casein	Alcalase	PPEIN, PLPLL	In vitro methods	[33]
Yak milk casein	Neutrase	YQKFPQY, LPQNIPPL, SKVLPVPQK, LPYPYY, FLPYPYY	In vitro methods	[34]
Yak hard chhurpi (cheese)	-	LYQEPVLGPVR	Molecular docking and BIOPEP database	[35]
Qula casein	Papain, Proteinase K, Trypsin, Alcalase, α-chymotrypsin, Thernolysin	KYIPIQ, LPLPLL, PFPGPIPN, QKEPMIGV	Molecular docking	[36]
Qula casein	Thermolysin + Alcalase T hermolysin + Proteinase K	KFPQY, MPFPKYP, MFPPQ, QWQVL	In vitro methods and molecular docking	[37]
Yak milk casein	Thermolysin + Papain	-	BIOPEP database	[38]

**Table 5:** Antioxidant activity of yak milk casein degradation peptides.

Source of Bio-Active	Used Enzymes	Peptide Sequence	Model/Method	Reference
Yak milk casein	Pepsin, Trypsin, Alcalase, Papain, Flavozyme	-	In vitro methods	[29]
Yak milk cheese	-	RPKHPIK	In vitro methods and molecular docking	[44]
Yak milk residues	Pepsin, Trypsin	KALNEINQF	H_2_O_2_-induced HUVECs	[45]
Yak milk casein	Alcalase, Trypsin	RELEEL, GKEKVNEL, LPVPQ, HPHPHL, VLPVP, VPYPQ	In vitro methods	[46]
Yak milk casein	-	AFK, IEQI, FPFF, LPVPQ, RELEEL	H_2_O_2_-induced HEK-293	[47]
Yak milk residues	Pepsin, Trypsin	MHQPHQPLPTVMF	H_2_O_2_-induced HUVECs and molecular docking	[48]
Fermented yak milk	-	ELPP, FDGDF, LYLKPR, LGDKKLF, KLLVAWVPP	H_2_O_2_-injured HT-22 cells and molecular docking	[49]

**Table 6:** Anti-inflammatory activity of yak milk casein degradation peptides.

Source of Bio-Active	Used Enzymes	Peptide Sequence	Model/Method	Reference
Yak milk casein	Pepsin, Trypsin, Alcalase, Papain, Flavozyme	-	Murine peritoneal macrophages	[29]

**Table 7:** Antidiabetic activity of yak milk casein degradation peptides.

Source of Bio-Active	Used Enzymes	Peptide Sequence	Model/Method	Reference
Yak milk cheese	-	RPKHPIK, KVLPVPQ	In vitro methods, Molecular docking, and BIOPEP database	[13]

**Table 8:** Antibacterial activity of yak milk casein degradation peptides.

Source of Bio-Active	Used Enzymes	Peptide Sequence	Model/Method	Reference
Yak milk cheese	-	RPKHPIK, TPVVVPPFL, VYPFPGPIPN, SLVYPFPGPIPN	Molecular docking and BIOPEP database	[2]
Yak butter	Pepsin	RVMFKWA, KVISMI	In vitro methods	[63]
Yak milk casein	Trypsin	-	In vitro methods	[64]
Yak milk casein	Flavozyme	APKHKEMPFPKYP, KEMPFPKYP, YPELFRQF	In vitro methods	[65]

**Table 9:** Anticancer activity of yak milk casein degradation peptides.

Source of Bio-Active	Used Enzymes	Peptide Sequence	Model/Method	Reference
Yak milk casein	Combined hydrolysis of trypsin and alkaline protease	TPVVVPPFL, VAPFPEVFGK, NQFLPYPY	Cell culture	[30]

**Table 10:** Immunomodulatory activity of yak milk casein degradation peptides.

Source of Bio-Active	Used Enzymes	Peptide Sequence	Model/Method	Reference
Yak milk casein	Alcalase	-	Murine T cells	[72]
Yak milk casein	Pepsin, Trypsin, Alcalase	-	Murine splenocytes	[73]
Yak milk casein	-	-	In vitro methods	[74]
